# Marginally scientific? Genetic testing of children and adolescents for lifestyle and health promotion

**DOI:** 10.1093/jlb/lsv038

**Published:** 2015-11-16

**Authors:** Timothy Caulfield, Pascal Borry, Maeghan Toews, Bernice S. Elger, Henry T. Greely, Amy McGuire

**Affiliations:** 1 Health Law Institute, University of Alberta, Edmonton, T6G 2H5, Canada; 2 Centre for Biomedical Ethics and Law, Department of Public Health and Primary Care, KU, B-3000 Leuven, Belgium; 3 Health Law Institute, University of Alberta, Edmonton, T6G 2H5, Canada; 4 Institute for Biomedical Ethics, Universität Basel, CH-4056 Basel, Switzerland; 5 Center for Law and the Biosciences, Stanford University, Stanford, CA 94305-8610, USA; 6 Center for Medical Ethics and Health Policy, Baylor College of Medicine, Houston, TX 77030, USA

**Keywords:** Direct-to-consumer genetic testing, genetic testing of adolescents, genetic testing of children, genetic testing policy, genetic testing regulation

## I. INTRODUCTION

*‘Genetic testing can reveal crucial information to guide your training and nutrition strategies based on predispositions built into your DNA. Genetic testing identifies the ideal nutrition, training and recovery strategies for you and helps explain why athletes given the same training and nutrition plans respond differently. In short gene testing provides the ultimate in personalized nutrition and training guidance…’*^*1*^

Direct-to-consumer (DTC) genetic testing has been in the news in recent years, especially since November 2013, when the US Food and Drug Administration (FDA) shut down 23andMe, one of several firms that offered people general health information based on their genetic test results.^2^ Much attention has been paid to the accuracy of these tests in predicting future risk of disease. This is understandable as the first wave of DTC companies, including 23andMe, eventually focused on providing genetic information of this nature. And much of the existing literature on the possible harms and benefits of DTC testing is largely an analysis of disease-oriented testing.^3^ But it should not be forgotten that many other DTC genetic testing products are being marketed throughout the world that are not focused on disease risk but instead offer information—of varying degrees of scientific legitimacy—relevant to things like fitness, diet, and athletic ability. In addition, non-traditional providers, such as homeopaths,^4^ naturopaths,^5^ and chiropractors,^6^ increasingly are offering genetic tests for the purpose of providing health and lifestyle advice.

It seems inevitable that, unless the regulatory environment changes, this trend will continue, with genetic testing being offered to the public for an ever-increasing range of reasons not directly related to disease. In this paper, we map the policy issues associated with the marketing and use of genetic testing products outside of the context of disease risk. We focus on companies and providers that offer testing for a wide range of genetic traits, and consider the potential implications of offering these products for use in children and adolescents. While the size of the market for these services remains unknown, it is probably still relatively small.^7^ Nonetheless, an analysis of genetic testing in this broader context provides an opportunity to consider issues that are likely to increase in significance as genetic testing technologies become more affordable and as the idea of genetic testing becomes more socially acceptable.^8^

For example, the present analysis affords an opportunity to consider the possible harms, if any, and available policy options for regulating genetic testing services not focused on disease risk and that reside on the margins of credibility. As we will see, some tests are being marketed for purposes that are clearly scientifically absurd (eg for targeting homeopathic therapies and naturopathic detoxification regimens)^9^ while others have more scientific plausibility, at least theoretically (eg testing for genes related to obesity and athletic characteristics like sprinting ability). Obviously, not all of these services will raise the same issues. But what they do have in common is the provision of genetic information. Are most of these companies and providers simply leveraging the excitement surrounding genetics to market services with unproven utility that are largely harmless? Is there something about genetic testing, as opposed to other forms of equally predictive information, which justifies heightened regulatory oversight?

Looking at these, and other,^10^ issues through the lens of children and adolescents highlight potential policy challenges that may be especially problematic.^11^ While one could argue that legally competent adults should have the right to choose whatever service they have an interest in (however useless), this may not be the case in the context of children and adolescents who lack legal capacity and may be particularly vulnerable to DTC industry marketing strategies. We will examine whether, in the context of genetic testing services that have little scientific data to support their purported uses, any regulatory or policy response is appropriate and the potential forms such a response could take.

## II. EXISTING POLICY ON TESTING OF MINORS

Numerous policy guidelines have addressed the genetic testing of minors in the clinical context. Most of these guidelines were crafted in response to the availability of disease and predisposition testing.

If a minor is suspected of having a condition where obtaining genetic information is considered clinically useful—be it for diagnostic or treatment purposes—testing is, in general, viewed as an appropriate course of action.^12^ However, many existing guidelines have stressed that predictive and pre-symptomatic genetic testing during childhood or adolescence should only be performed for conditions for which preventive or therapeutic actions could and should be initiated.^13^ The underlying rationale is that minors should benefit directly from testing. In the absence of obvious health benefits, support for genetic testing of minors erodes, largely because of the belief that individuals should be able to make autonomous decisions about genetic testing when competent adults and that testing may have an unknown psychosocial impact.^14^

Given these concerns, it is not a surprise that most clinical guidelines and position papers are also critical of DTC genetic testing companies that test minors. The most recent guideline on the testing of minors was published by the American Academy of Pediatrics and the American College of Medical Genetics and Genomics. In their statement, both societies ‘strongly discourage the use of direct-to-consumer and home kit genetic testing of children because of the lack of oversight on test content, accuracy, and interpretation’.^15^ In the technical report supporting the recommendation, Ross et al. refer to the risks of inaccurate results, inaccurate interpretations, potentially harmful interventions, issues of privacy, self-determination, and (non-) disclosure, as well as to the importance of professional involvement in any type of genetic testing on minors.^16^ Similar and additional concerns have been raised in other guidelines and position papers on DTC genetic testing,^17^ including the European Academies of Science Advisory Council, the Federation of European Academies of Medicine, and the European Society of Human Genetics.^18^

Existing policy documents, however, also note that not all concerns apply equally to all types of tests. The UK Human Genetics Commission, for example, acknowledged the importance of considering both the type of test provided and the impact of that test. In situations where tests could have a ‘significant or potentially detrimental impact on consumers’ appropriate support and professional advice is more important than in situations where testing might not create that type of impact.^19^ This also has consequences for genetic testing in minors, where the Human Genetics Commission would limit genetic testing in children for diagnostic tests, pre-symptomatic tests, carrier tests, susceptibility tests, and pharmacogenomics tests, but is not against allowing nutrigenomic tests and lifestyle/behavioral tests that have not been evaluated as ‘high impact’.^20^ Similarly, the Canadian College of Medical Geneticists described rather clearly that their statement applies to ‘medically significant genetic testing’ and stated in this line that ‘professional guidelines related to the practice of medical genetics should be adhered to, particularly with respect to genetic testing of children’.^21^

This distinction between ‘medically significant’ genetic tests on one hand and lifestyle-oriented, recreational, or informational types of genetic tests on the other hand creates room for a policy debate. Do these latter types of services that are aimed at issues not tied directly to health—such as athletic ability—or do not relate to something of immediate clinical relevance—such as diet—but that are often based on less-than-robust science, give rise to similar potential harms as ‘medically significant’ tests? Are the potential harms significant enough to trigger regulatory oversight?

Most policy debates have focused on tests pertaining to predisposition to disease offered by DTC companies such as 23andMe and there has been little attention, at least from a policy perspective, on these more lifestyle-oriented genetic testing services. Indeed, many of the policy responses, such as those by professional societies, seem to assume, without much, if any, discussion, that there is potential for real clinical or health relevance, which requires oversight. Below we review in greater detail some of these ‘lifestyle’ types of tests and the potential policy issues they raise, especially when made available for use in children and adolescents.

## III. EXAMPLE ‘LIFESTYLE’ SERVICES

### (i) Athletic ability

Companies throughout the world offer genetic testing for the purpose of assessing athletic ability. Atlas Sports Genetics, for example, provides a testing service that promises to ‘show athletes, trainers and interested individuals where their genetic advantage lies’.^22^ A UK company, DNA Fit, provides a test that will allow you to ‘explore your natural ability’.^23^ And Gonidio promises to ‘Identify your athletic strengths and weaknesses!’ and allows consumers to ‘Choose a sport that suits you!’^24^ The premise behind these companies is straightforward: get your genes tested and uncover your specific athletic potential (or lack thereof).

We know of no evidence about how many minors (or their parents) are using these services.^25^ Indeed, an analysis of the marketing strategies of these companies found that they generally do not explicitly market their services to parents and kids,^26^ although some companies do specifically market to parents, encouraging them to test their children in order to discover their ‘inborn talents’, including—among other things—athletic abilities.^27^ Additionally, there have been reports of plans to use the technology on children in order to facilitate ‘sports selection at the molecular genetic level’.^28^ That said, it seems reasonable to assume that there is a potential market in this area.^29^ Many families invest huge sums in the sport activities of their children. They pay for expensive lessons and training programs, all in the hope of boosting their children's athletic careers. The cost to have one child play high-caliber minor hockey in Canada ranges from $8000 to $15,000 per year.^30^ The cost of having a child train as an aspiring Olympian can be even more significant. It has been estimated that competitive gymnastics, for example, averages about $15,000 per year.^31^ Sport is often a family commitment and the pressure to perform can come from the parents.^32^ Given this context, there seems a real possibility that some parents will want to maximize their child's odds at achieving athletic success through genetic testing.^33^

In addition, abundant evidence shows that adolescents will often go to great lengths to gain a competitive edge in their chosen sport. Given that a significant portion of teenagers are willing to take illegal performance enhancing drugs,^34^ getting a genetic test seems well within the scope of strategies some teenagers may pursue. Finally, for many of the sports associated with the athletic DTC genetic testing, such as American football and sprinting, testing must occur at a relatively young age if the results are to be used, as suggested by the associated marketing, to craft and focus an athletic career. For these sports, starting as a youth is crucial. Few people, for example, start playing football as adults.^35^ As one company notes, ‘the earlier the child starts, the easier it is to get a headstart over other children’.^36^ Indeed, the idea of exploring whether we are ‘better suited to sports requiring endurance, strength, or speed’^37^ seems a question largely aimed at individuals at the beginning of their athletic careers. So while these companies may not all direct their marketing at youth (or their parents), this is the demographic that should be most interested in using these services.

The actual performance value of these tests, if any, is far from clear. Can these DTC genetic testing services really provide useful, actionable information? The existing evidence on point can, at best, be described as equivocal. There is absolutely no doubt that genes play an important part in athletic performance.^38^ Particular aptitudes—such as speed and endurance—have been associated, though usually weakly, with particular polymorphisms.^39^ Yet, many announced associations between genomic variations and traits or even diseases have not been replicable. The relationship between genes and future athletic performance is far from clear.^40^ Indeed, most agree that athletic success results from a complex combination of genes, epigenetics, personality, psychological competences, training, and a host of other environmental factors (eg the luck to have been raised in a supportive environment).^41^ As a result, these tests are unlikely to predict whether a given child or adolescent will blossom into an elite sports star.^42^ Most of the scientific literature on point questions the value of the testing technology as a way to predict future performance.^43^ Indeed, as summarized in a recent review of the relevant science, ‘few genes are consistently associated with elite athletic performance, and none are linked strongly enough to warrant their use in predicting athletic success’.^44^ It is also true that in many situations, an excellent and inexpensive way to test a child for, say sprinting versus distance running, is to watch her run. As with all complex human behaviors, it seems a mistake to think that genetic testing should be used to shape the athletic future of children and adolescents.

### (ii) ‘Healthy living’ testing

One of the core concepts behind personalized medicine is that genetic testing can be used to tailor our lifestyles in order to maximize health promotion.^45^ DTC genetic testing services are marketed as a way to improve things like diet, exercise, and screening practices. Indeed, the idea of personalized lifestyle advice is central to most DTC genetic services and virtually all DTC genetic testing companies and alternative medicine providers offering these types of tests push the idea that their service can be used to individualize specific interventions to optimize healthy living.^46^ Apart from tests for disease risk, these ‘healthy living’ genetic tests are being offered for a wide range of traits including weight, diet, food intolerance, eating behavior, and drug responses.^47^ In the context of tests being marketed directly for use in children and adolescents, additional traits purportedly tested for include sensitivity to second-hand smoke, alcoholism, general addiction, general wellness, hyperactiveness, ‘propensity for teenage romance’, ability to focus, nearsightedness, and whether the child is a morning or night person.^48^

In addition to being marketed directly to consumers, genetic testing services are also being offered by a range of health care practitioners, such as naturopaths,^49^ homeopaths,^50^ and chiropractors.^51^ Indeed, some DTC companies market their testing services to alternative providers, encouraging them to make genetic testing part of their ‘personalized’ approach.^52^ It is no surprise, then, that these alternative practitioners—a group that likely has little or no formal training in genetics^53^—cast the services as an important component of an overall, holistic approach to health and as a way to personalize lifestyle interventions. One naturopathic clinic, for example, suggests that its genetic testing service will ‘allow you to influence your genetic variant using specific food, lifestyle and exercise’.^54^

Once again, it is unclear how big the genetic testing market is for these ‘healthy living’ type of services or how many children and adolescents are being tested. But it is worth noting that there are some musings in both the academic and popular media about testing children for a genetic predisposition to obesity.^55^ And research on parental attitudes has found that most feel comfortable testing children and would like an obesity test to be available.^56^ Given that some parents will go to great lengths to optimize the health of their children, it seems reasonable to conclude that children and adolescents are being or soon will be tested for the purpose of lifestyle and health promotion. Several companies are now offering genetic testing services for obesity predisposition that are targeted directly for use in children^57^ and another company suggests testing ‘should be considered for children who are over the age of 14’.^58^

Can these tests really provide useful information that will change behavior? Is a personalized approach really more effective?^59^ To date, despite the significant push for personalized medicine,^60^ there is very little evidence to support the idea that a personalized and genetically informed approach is any better than general health and lifestyle advice, be it in the context of nutrition, exercise, or weight loss.^61^

First, the associations being tested are usually very weak. Even the most scientifically valid genetic tests are far from being highly predictive of, for example, a future risk of obesity.^62^ The recommendations being made on the basis of these tests by many companies not only lack supportive evidence but in some cases are scientifically absurd. For example, one clinic suggests that the testing will reveal if you require more ginseng.^63^ Others claim that we can use genetic testing to fine-tune the power of a homeopathic remedy, which again has no basis in science.^64^

Second, there is no evidence that people use this information to make behavioral changes.^65^ Indeed, the best available evidence suggests that genetic risk information does not lead to behavior change.^66^ This evidence largely comes from studies of adults, however, and it is unclear whether children will be more malleable or whether parents may be more motivated for behavioral change when it comes intervening in their children's lives than their own.

## IV. POTENTIAL HARMS

Given the questionable scientific validity or predictive power of many of these testing services and the lack of any evidence of clear health benefit, what are the harms of testing children and adolescents in this context and are they significant enough to warrant a policy response?

### (i) Psychological or physical harms

Concerns exist about direct psychological or physical harms to children that could result from genetic testing. In the context of ‘healthy living’ tests, it is possible that parents purchasing these tests to use on their children could have misunderstandings about the validity and relevance of these tests which could lead to harmful interventions. For example, testing children for obesity or their ability to tolerate second-hand smoke might lead to physical harm if parents unnecessarily restrict their children's diets^67^ or expose them to second-hand smoke. Children and adolescents who undergo testing on their own initiative may also exhibit these types of behaviors and grow up misinformed about their genetic information and the significance (or lack thereof) of their genetic test results.

There is also the possibility that children or adolescents might face subtle forms of stereotyping from parents, coaches, schools, peers, and others based on the mistaken belief that the information is more powerful than it is.^68^ Athletic testing—whether pursued by parents or children themselves—could, for instance, discourage parents from supporting and children from pursing a particular sports interest if the genetic test does not confirm specific talents and could therefore cause harm by overall decreasing sports activities. It is also possible that children or parents could make life decisions about the children based on mistaken beliefs about the relevance of these tests. This could lead to disappointment or anxiety in children whose apparent genetic make-up fails to align with their (or their parents’) existing interests or desires.^69^

While studies examining how people actually react to genetic information regarding predisposition for disease show that receiving this information generally does not have a significant effect on people's psychological outcomes (eg it does not result in significant anxiety or depression),^70^ it is unclear whether this finding extends to the psychological impact on children of genetic testing for athletic ability or healthy lifestyle. This may be particularly difficult to determine as the impact of testing on children—including the impact of altered parental expectations and support—may be more subtle and nuanced than what is captured in most measures of psychological outcomes. Indeed, evidence is insufficient to understand how children and their parents react and respond to genetic testing more generally.^71^ The extent to which decisions regarding the desirability of DTC genetic testing of children can be guided by actual evidence, as opposed to speculation about potential harm, is therefore open to debate.

### (ii) Autonomy

As noted above, parental genetic testing of children raises concerns about respect for children's autonomy, including the decision to pursue testing when they reach the age of capacity. In clinical genetic testing, the norm is to let children exercise their autonomy by making choices when adults about conditions that do not affect children. Whether the reasons for this position exist to the same extent in relation to athletic and ‘healthy living’ types of genetic testing is less clear.

On one hand, the fact that the information provided by many of these tests is poorly predictive and of no real benefit may weigh in favor of preserving a child's ability to decide whether or not to undergo genetic testing when the child has sufficient capacity. On the other hand, given the poor quality of information that is often provided it is questionable in some cases how much genetic information is actually being disclosed. For example, test results indicating a strong ‘propensity for teenage romance’ or poor ‘general wellness’ are not likely a reflection of a child's actual genetic make-up. The harm here appears to be more a problem of misleading claims in the marketing and advertising of these tests. To the extent that the information from these tests limits a child's or adolescent's ability to make decisions that are consistent with their goals, however, there may nevertheless be an autonomy concern. This concern may be more pronounced in some tests, such as many of the athletically focused tests, which actually look for specific variants of a particular gene and have the potential to limit a child's ability to explore different sports and activities. However, the question then becomes whether disclosing a child's genetic predisposition for endurance sports, for example, warrants the same kind of protection as disclosing a child's genetic predisposition for developing disease.

If an individual's genetic information in and of itself, regardless of its lack of clinical relevance, is something that only autonomous individuals should have access to and control of, then perhaps all forms of athletic and ‘healthy living’ genetic testing of children by parents should be prohibited. However, unless something about genetic information is inherently different, perhaps inherently more convincing, it is difficult to rationalize why a genetic indicator of a child's sprinting ability, for example, should be prohibited while empirical indicators (such as timing the child while running, which is likely a more predictive test) should not.

In the clinical genetic testing context, what's at stake is the child's ability to decide whether or not to discover his or her genetic likelihood of developing future health problems. In the context of an athletic or ‘healthy living’ genetic test that does not provide any clinically relevant or meaningful health information, the impact of the information will rarely if ever reach that level.

It is also important to consider the autonomy interests of the child or adolescent who wants to undergo genetic testing and the potential for restrictive policies to interfere with a child's right to know his or her personal genomic information. Should the choice to undergo athletic and ‘healthy living’ genetic testing be treated like a medical decision where a child or adolescent has to reach a certain level of capacity to be able to consent? Or should these tests be treated like any other consumer product, and if so should there be age restrictions for access as is the case with alcohol, tobacco, and pornography? Age restrictions on the ability to purchase these products exist because there are demonstrated health risks and social harms associated with their use. In the case of DTC genetic tests where proof of harm remains lacking, it is less clear whether children and adolescents should be prohibited from purchasing these testing services if they so wish.^72^ Would it be appropriate, for example, to craft a policy that would prevent a child or a child's parent from wasting money on a horoscope or a tarot card reading?

### (iii) Financial exploitation—‘Scienceploitation’

The lack of any demonstrated benefit for these types of athletic and ‘healthy living’ tests certainly suggests that charging money for them—either to parents or children and adolescents themselves—under the auspices of providing useful information is financially exploitative. Many of these companies seem to be simply leveraging the excitement surrounding genetics to market products^73^—at times throwing in references to genetics to heighten the scientific legitimacy of the service, even when it isn't clear if it is an actual genetic test.^74^ At a minimum, this issue points to the importance of accurate information, the need to control the marketing of these tests, and the role of educating parents and child/adolescent consumers about the actual relevance and utility of these tests.

## V. POLICY IMPLICATIONS

While few would dispute the idea that these kinds of athletic and ‘healthy living’ genetic tests raise social issues, are the potential harms significant enough to trigger a regulatory response? For example, in the absence of evident harms^75^ parents are generally free to take their children to see astrologers, homeopaths, or naturopaths, which reside outside or on the margins of scientific credibility and many alternative practitioners provide health advice that sounds scientific but is, in fact, not supported by available evidence or even the basic principles of science (eg allergy and food intolerance testing by naturopaths).^76^ Is genetic testing in this context any worse or better?

Some European countries have taken a restrictive stance on genetic testing more generally with legislation that limits or prohibits DTC genetic testing.^77^ Under a very cautious approach where, in the absence of proven benefit, even the speculative possibility of harm is something to be guarded against, legislation of this nature may be useful to limit companies and providers from operating or making these athletic and ‘healthy living’ genetic testing products available to children and adolescents in a particular jurisdiction. However, there are ongoing debates about the scope of these pieces of legislation, whether they prohibit all forms of DTC genetic tests, and whether further legislative change in Europe is on the horizon.^78^ Moreover, there may be issues with enforcement given that DTC genetic testing companies operate online and do not need to establish a physical presence in a given jurisdiction to sell or deliver products to consumers who reside there. Additional restrictions targeting the actual use of these products by consumers may be useful in increasing the effectiveness of this approach. For example, regulatory bodies could seek to limit the marketing and use of these services by health care providers. Moreover, age limits could be set establishing a minimum age to be tested or to purchase a test. However, it is debated—in particular in non-European countries—whether such a prescriptive approach is warranted given the lack of evidence of direct harm.^79^

There may also be a role for health authorities and regulatory agencies to address these genetic tests. The FDA in the United States, for example, asserted its jurisdiction over 23andMe's test for disease predisposition on the basis that this test was a ‘diagnostic device’.^80^ To the extent that some of the ‘healthy living’ genetic tests offer information on disease-risk, drug response, or other medically relevant traits, it is possible that they may also come within the FDA's jurisdiction to regulate diagnostic and other types of medical devices. In this regard, the FDA could take action similar to its response to 23andMe by prohibiting companies from offering genetic tests that have not received appropriate marketing authorization. In addition, the FDA in some cases can regulate the age at which consumers can purchase certain products, such as tobacco products, as well as the marketing of these products to limit children's exposure to advertising.^81^ If the FDA viewed genetic testing products as posing particular risks to children and adolescents, it could potentially use a similar regulatory tool to address the marketing and consumption of these products.

However, it is less likely that athletic DTC genetic tests or some of the ‘healthy living’ tests with less clinical relevance would similarly come within the FDA's jurisdiction. Additionally, it is unlikely that the analogous regulatory bodies in Canada or the United Kingdom would have jurisdiction over any of these tests as these bodies have taken the position that although DTC genetic testing kits themselves may fall within their respective jurisdictions the actual testing *service* does not.^82^ As a result, 23andMe is able to operate legally in both these jurisdictions and it is unlikely that a stronger stance would be taken by these bodies against companies offering athletic and ‘healthy living’ types of tests, which have even less direct health relevance.

As many of the potential harms associated with these types of genetic tests pertain to a lack of accurate information and understanding about the validity and relevance of the genetic information provided, there may be a role for regulatory bodies responsible for enforcing truth in advertising standards. These bodies address complaints about potential misrepresentations in advertisements and have the authority to limit the types of representations that companies can make. For example, the US Federal Trade Commission (FTC) recently took action against GeneLink, Inc., which offered genetic testing in conjunction with the sale of nutritional supplements that were purportedly tailored to its customers’ DNA profiles. The FTC issued an order prohibiting GeneLink from making any representations ‘about the health benefits, performance, or efficacy’ of its product ‘unless …[GeneLink, Inc.] possesses and relies upon competent and reliable scientific evidence that is sufficient in quality and quantity based on standards generally accepted in the relevant scientific fields’.^83^ It seems that similar complaints could be brought against many of the other DTC genetic testing companies offering athletic and ‘healthy living’ tests.

In addition, it may be useful to provide educational interventions aimed at physicians or the general public about the lack of scientific validity and relevance of many of these tests. There is evidence that consumers of disease-risk DTC genetic testing sometimes consult health care providers about test results.^84^ However, it is unclear whether consumers of athletic and ‘healthy living’ types of genetic tests would be equally likely to consult their providers about these kinds of test results, which may limit the effectiveness of this intervention. In addition, there may be value in providing accurate information to parents contemplating these tests for their children or to children and adolescents themselves who may be considering genetic testing.

Finally, future research should focus on evaluation of the more subtle and nuanced benefits and harms of athletic and ‘healthy living’ genetic testing of children and adolescents. Socially relevant outcomes would include impact on self-esteem, feelings of vulnerability, and experiences of social stigma and discrimination, among others. The lack of sufficient evidence of the benefits and harms of these services highlights the need for further empirical research to guide policy development.

## Footnotes

1 Institute for Optimum Nutrition, *Genetics & Performance: DNA Testing Service*, http://www.ion.ac.uk/performance/dna_testing (accessed June 16, 2015).

2 See Food and Drug Administration, *Warning Letter to 23andMe*, Nov. 22, 2013, http://www.fda.gov/iceci/enforcementactions/warningletters/2013/ucm376296.htm (accessed June 18, 2015). Although the FDA prohibited 23andMe from continuing to offer its DTC genetic test regarding predisposition to disease, the FDA has recently permitted 23andMe to market a specific DTC genetic carrier test for Bloom syndrome. See FDA News Release, *FDA Permits Marketing of First Direct-to-Consumer Genetic Carrier Test for Bloom Syndrome*, Feb. 19, 2015, http://www.fda.gov/newsevents/newsroom/pressannouncements/ucm435003.htm (accessed June 18, 2015).

3 See, for example, Timothy Caulfield & Amy McGuire, *Direct-to-Consumer Genetic Testing: Perceptions, Problems and Policy Responses*, 63 Annu. Rev. Med. 23 (2012).

4 Natural Health Ministries, *Homeopathic Energy DNA Testing*, www.naturalhealthministries.com/index.php?page=homeopathic-energy (accessed June 16, 2015); Greenhouse Health, *Genetic Tests*, www.greenhousehealth.com/tests/genetic-tests-2/ (accessed June 16, 2015).

5 For example, see Brunswick Health, *DNA Testing*, www.brunswickhealth.com.au/dna-testing.html (accessed June 16, 2015), which markets its products by stating that customers can ‘[d]iscover how measuring your genetic profile can lead to Nutritional and lifestyle changes that can target disease prevention and help you achieve optimal health’. See also Nature's Intention Naturopathic Clinic, *Your Genetic Testing Center*, http://www.naturesintentionsnaturopathy.com/genetic-testing/toronto-genetic-testing-centre.htm (accessed Nov. 2, 2014).

6 For example, Ingram Health Care P.C., *DNA Testing*, www.ingramhc.com/dna-testing.html (accessed June 16, 2015); Town Center Wellness, *Functional Nutrition*, www.towncenterwellness.com/services/nutrition/ (accessed June 16, 2015); and Dr. Jess, *Genetic Testing for Sports Injuries*, Inner Sport Chiropractic, Aug. 12, 2013, www.innersport.com/archives/2421 (accessed June 16, 2015).

7 Caroline F. Wright & Shelley Gregory-Jones, *Size of the Direct-to-Consumer Genomic Testing Market*, 12Genet. Med. 594 (2010). Indeed, as noted by Art Wuster, *Is Direct to Consumer Genetic Testing Profitable?*, Sequonomics, Oct. 6, 2013, www.seqonomics.blogspot.co.uk/2013/10/is-direct-to-consumer-genetic-testing.html (accessed June 16, 2015), many of the most well-known companies—such as Navgenics, deCodeMe, and Pathway Genomics—have discontinued or greatly narrowed their services. See also Philippa Brice, *Current DTC Genetic Testing Market Smaller than Expected*, Phg Foundation, Aug. 30, 2010, www.phgfoundation.org/news/5658/ (accessed June 16, 2015).

8 Juli M. Bollinger, Robert C. Green & David J. Kaufman, *Attitudes About Regulation Among Direct-to-Consumer Genetic Testing Customers*, 17 Genet. Test. Mol. Biomarkers 424 (2013).

9 See, for example, Peter Kay, *Significance of the Sequence Specific Homeopathic DNA Therapy System in Modern Healthcare*, Positive Health Online, June, 2013, http://www.positivehealth.com/article/dna-gene-expression/significance-of-the-sequence-specific-homeopathic-dna-therapy-system-in-modern-healthcare (accessed June 16, 2015), and the website for Naturopathic Doctors, *YouNique Genomics—Genetic Testing*, http://thenaturedoctors.ca/services/specialty-testing/younique-genomics-genetic-testing/ (accessed June 16, 2015).

10 For example, there are DTC genetic testing companies offering ancestry testing, which may have unanticipated implications for consumers (eg discovering their family members is not genetically related to them or that they have siblings or other relatives they never knew about). See George Doe, *With Genetic Testing, I Gave My Parents the Gift of Divorce*, Vox Genetics, Sept. 9, 2014, http://www.vox.com/2014/9/9/5975653/with-genetic-testing-i-gave-my-parents-the-gift-of-divorce-23andme (accessed June 16, 2015), and Julia Belluz, *Genetic Testing Brings Families Together: And Sometimes Tears Them Apart*, Vox Genetics, Dec. 18, 2014, http://www.vox.com/2014/9/9/6107039/23andme-ancestry-dna-testing (accessed June 16, 2015).

11 Pascal Borry et al., *Direct-to-Consumer Genome Scanning Services. Also for Children?*, 10 Nat. Rev. Genet. 8 (2009).

12 Mary E. Fallat et al., *Ethical and Policy Issues in Genetic Testing and Screening of Children*, 131 Pediatrics 620 (2013). As recently stated by the American Academy of Pediatrics and the American College of Medical Genetics and Genomics, ‘[i]n a child with symptoms of a genetic condition, the rationale for genetic testing is similar to that of other medical diagnostic evaluations. Parents or guardians should be informed about the risks and benefits of testing, and their permission should be obtained. Ideally and when appropriate, the assent of the child should be obtained’.

13 Pascal Borry et al., *Presymptomatic and Predictive Genetic Testing in Minors: A Systematic Review of Guidelines and Position Papers*, 70 Clin. Genet. 374 (2006). For example, the German Society of Human Genetics stated that ‘Predictive diagnosis may be carried out for persons of legal age only, except for disorders in which important therapeutic or preventive measures could be initiated in childhood’. Committee for Public Relations and Ethical Issues of the German Society of Human Genetics, *Position Paper of the German Society of Human Genetics*, 8 Med Genetik 125 (1996). As well the Danish Council of Ethics has stated that testing in minors should only be performed if testing ‘provides scope for treatment which to any essential degree prevents, defers or alleviates the outbreak of disease or the consequences of the outbreak of disease’. Danish Council of Ethics, *Genetic Investigation of Healthy Subjects—Report on Presymptomatic Gene Diagnosis*, 2001, http://www.etiskraad.dk/sw293.asp (accessed June 16, 2015).

14 The European Society of Human Genetics has recommended that testing in minors ‘should be delayed until the person is old enough to make an informed choice’. European Society of Human Genetics, *Genetic Testing in Asymptomatic Minors: Recommendations of the European Society of Human Genetics*, 17 Eur. J. Hum. Genet. 720 (2009). Note, there is increasing recognition of an exception to this principle where a child's genetic test reveals incidental findings about adult onset disease and that information is informative about the parents’ and other family members’ risk of disease in which case disclosure would benefit the family. See Robert C. Green et al., *ACMG Recommendations for Reporting of Incidental Findings in Clinical Exome and Genome Sequencing*, 15 Genet. Med. 565 (2013). See also Amy L. McGuire et al., *Ethics and Genomic Incidental Findings*, 340 Science 1047 (2013).

15 Fallat et al., *supra* note 12.

16 Laine F. Ross et al., *Technical Report: Ethical and Policy Issues in Genetic Testing and Screening of Children*, 15 Genet. Med. 234 (2013).

17 For example, the Austrian Bioethics Committee refers to the fact that persons can be tested without their knowledge and consent and that children should be protected in that respect: Austrian Bioethics Commission, *Report of the Austrian Bioethics Commission on Internet-Based Genetic and Genome-Wide Testing*. May 10, 2010 http://www.bka.gv.at/DocView.axd?CobId=40383 (accessed June 16, 2015).

18 European Academies Science Advisory Council and the Federation of European Academies of Medicine, *Direct-to-Consumer Genetic Testing for Health-Related Purposes in the European Union*, Easac Policy Report (2012), ‘Testing of samples from minors and third parties should not be permitted’. European Academies of Science Advisory Council and the Federation of European Academies of Medicine; and European Society of Human Genetics, *Statement of the ESHG on Direct-to-Consumer Genetic Testing for Health-Related Purposes*, 18 Eur. J. Hum. Genet. 1271 (2010). Most of these documents focus on concerns related to the weakness of the scientific evidence supporting certain tests, their lack of clinical utility, the inappropriate labeling and advertisements of tests, the provision of inappropriate information, the lack of informed consent, and the absence of genetic counseling or medical supervision.

19 Human Genetics Commission. *A Common Framework of Principles for Direct-to-Consumer Genetic Testing Services*, 2010, http://www.sashg.org/documents/HGC-UK-Policy-on-DTC-testing.pdf (accessed June 19, 2015).

20 *Id.* at 10. The Human Genetics Commission in the UK advanced that genetic testing in situations where a child does not have capacity to consent should normally be deferred until the attainment of such capacity, unless other factors indicate that testing during childhood is clinically indicated. If postponement would be detrimental to the child's health, or the management of the child's health may be altered significantly depending on the test result, then testing should be organized by a health professional who has responsibility for ensuring that any medical intervention or screening indicated will be arranged and proper arrangements made for any subsequent care.

21 Tanya N. Nelson et al., *CCMG Statement on Direct-to-Consumer Genetic Testing*, 81 Clin. Genet. 1 (2012).

22 ATLAS Sports Genetics, http://www.atlasgene.com/ (accessed June 16, 2015).

23 DNAFit, http://www.dnafit.com (accessed June 16, 2015).

24 Gonidio, *Athletic Performance*, http://www.gonidio.com/test.php?id=2 (accessed June 18, 2015).

25 While we could find no obvious age restrictions on the websites, past research has disclosed recommended ages. See Jennifer K. Wagner & Charmaine D. Royal, *Field of Genes: An Investigation of Sports-Related Genetic Testing*, 2J. Pers. Med. 119, 121 (2012): ‘[A] few companies provided recommended ages for their products, such as Athleticode (not intended for individuals under 13 years), Atlas Sports Genetics (not intended for individuals under 10 years), and My Gene (not intended for individuals under 18 years)’.

26 Wagner & Royal, *supra* note 25 at 131: ‘Marketing strategies were not found to be directed at parents to identify the next superstar athlete or determine how children should be raised’.

27 For example, Map my Gene, *Inborn Talent Genetic Test*, http://www.mapmygene.com/inborn.htm (accessed June 16, 2015) offers ‘The Inborn Talent Genetic Test’ that claims to identify genetic traits for endurance, sprint, technique, training sensitivity, tendency of sport injuries, and sport psychology. OOGene, *FAQ*, http://oogene.com/faq.html (accessed June 16, 2015) offers a genetic test to determine a child's innate potential for a variety of talents including ‘dancing abilities’.

28 See Ron Synovitz & Zamira Eshanova, *Uzbekistan Is Using Genetic Testing to Find Future Olympians*, The Atlantic, Feb. 6, 2014, where it is reported that ‘Uzbekistan hopes to get a jump on the competition by testing children as young as 10 to determine their athletic potential’. The program will be run in ‘cooperation with the National Olympic Committee’, http://www.theatlantic.com/international/archive/2014/02/uzbekistan-is-using-genetic-testing-to-find-future-olympians/283001/ (accessed September 10, 2015).

29 There are no figures available on the size of this market. However, the profile of these types of services seems to be increasing. See, for example, Anna White, *Latest UK Fitness Fad May Get Cameron Back into his Genes*, The Telegraph, Aug. 23, 2013, http://www.telegraph.co.uk/finance/comment/citydiary/10261279/Latest-UK-fitness-fad-may-get-Cameron-back-into-his-genes.html (accessed September 10, 2015).

30 CBC News, *Hockey, Canada's Game, Not its Most Popular: Hockey By the Numbers*, Cbc News, Sept. 20, 2013, http://www.cbc.ca/news2/interactives/sports-junior/ (accessed June 18, 2015).

31 Tom Van Riper, *The Cost of Raising a Summer Olympian*, Forbes, July 24, 2012, http://www.forbes.com/sites/tomvanriper/2012/07/24/the-cost-of-raising-a-summer-olympian/ (accessed June 18, 2015).

32 Eddie Brummelman et al., *My Child Redeems My Broken Dreams: On Parents Transferring Their Unfulfilled Ambitions onto Their Child*, 8 Plos One 1 (2013). See also Sebastiano Costa et al., *Perceived Parental Psychological Control and Exercise Dependence Symptoms in Competitive Athletes*, 13 Int. J. Mental Health Addiction 59 (2014).

33 Jennifer K. Wagner, *Playing with Heart and Soul…and Genomes: Sports Implications and Applications of Personal Genomics*, 1 Peer J. e120 (2013).

34 Recent research has found that, in the USA, approximately 7 per cent of teens use steroids and an even higher percentage use human growth hormone. See Partnership for Drug-Free Kids, *2013 Partnership Attitude Tracking Study, Sponsored by MetLife Foundation*, http://www.drugfree.org/wp-content/uploads/2014/07/PATS-2013-KEY-FINDINGS1.pdf (accessed June 17, 2015).

35 It is worth noting that all the websites have images of individuals playing sports like American football. And Gonidio, *supra* note 24, has an image of a child dunking a basketball.

36 OOGene, *supra* note 27.

37 Gonidio, *Sports Performance*, http://www.gonidio.com/sportsperformanceBROCHURE_english.pdf (accessed June 18, 2015).

38 Keith Davids & Joseph Baker, *Genes, Environment and Sport Performance: Why the Nature-Nurture Dualism is No Longer Relevant*, 37 Sports Med. 961 (2007); Nir Eynon et al., *Genes and Elite Athletes: A Roadmap for Future Research*, 589 J. Physiol. 3063 (2011); Ross Tucker, Jordan Santos-Concejero & Malcolm Collins, *The Genetic Basis for Elite Running Performance*, 47 Brit. J. Sports Med. 545 (2013).

39 See, for a recent example, Leysan J. Mustafina et al., *AGTR2 Gene Polymorphism is Associated with Muscle Fibre Composition, Athlete Status and Aerobic Performance*, 99 Exp. Physiol. 1042 (2014).

40 National Health and Medical Research Council, *Use of Genetic Information in Sport*, https://www.nhmrc.gov.au/health-topics/genetics-and-human-health/genetic-testing-and-applications/use-genetic-information-spo (accessed June 17, 2015): ‘It is unclear whether testing for this gene would be useful in making decisions about talent identification or even personalized training. Attempts by some football clubs in Australia to use genetic information for selection or training have not produced any reported benefits and were abandoned’.

41 See, generally, Ross Tucker & Malcolm Collins, *What Makes Champions? A Review of the Relative Contribution of Genes and Training to Sporting Success*, 46 Brit. J. Sports Med. 555 (2012). See also Martinus Buekers, Pascal Borry & Paul Rowe, *Talent in Sports. Some Reflections about the Search for Future Champions*, 88 Mov. Sport Sci. 3 (2014).

42 As nicely summarized in a quote by David Eptein, author of The Sports Gene (2013), ‘We have no clue what most genes do. You would have a better prediction by using a stopwatch and timing someone’, in Synovitz & Eshanova, *supra* note 28.

43 See Sigal Ben-Zaken et al., *Genetic Score of Power-Speed and Endurance Track and Field Athletes*, 25 Scand. J. Med. Sci. Sports 166 (2013): ‘We conclude that polygenetic scores can differentiate power-speed from endurance athletes. Whether these scores may be used to identify elite power-speed or endurance athletes’ needs to be addressed in future studies’. See also Roger Collier, *Genetic Tests for Athletic Ability: Science or Snake Oil?*, 184 Cmaj E43 (2012). It should be noted that there is a good deal of diversity is what is actually offered by the DTC industry. See, for example, Wagner, *supra* note 33 at 12, who found ‘considerable diversity in offerings and that there simply is no “typical” or “standard” sports-related DTC panel for the PGx industry’.

44 Lisa M. Guth & Stephen M. Roth, *Genetic Influence on Athletic Performance*, 25 Curr. Opin. Pediatr. 653 (2013). See also Hans-Joachim Appell Coriolano & Jose A. Duarte, *Studies on Gene Polymorphisms in Sports Fancy Fashion or Important Field of Research?*, 33 Int. J. Sports Med. 419 (2012): the genetics ‘studies demonstrate widely incongruent results’; and Amaya Buxens et al., *Can We Predict Top-Level Sports Performance in Power vs Endurance Events? A Genetic Approach*, 21 Scand. J. Med. Sci. Sports 570 (2011). Athletic traits are ‘likely not reducible to defined genetic polymorphisms’.

45 For a general review of this trend, see Timothy Caulfield, *The Obesity Gene and the (Misplaced) Search for a Personalized Approach to our Weight Gain Problems*, 5 Wake Forest J. L. Pol'y 125 (2015).

46 See Sheryl Ubelacker, *U.S. Company Launches Genetic Health and Ancestry Info Service in Canada*, Metro News, Oct. 1, 2014, http://metronews.ca/news/canada/1171060/u-s-company-offers-canadians-genetic-info/ (accessed June 17, 2015), which notes that ‘a key goal of 23andMe is providing DNA-based information that could encourage people to improve their lifestyles in order to prevent diseases for which they may have an inherited predisposition’. See also Brunswick Health, *supra* note 5; Nature's Intentions Naturopathic Clinic, *supra* note 5; and Dr. Jess, *supra* note 6.

47 See Home DNA Direct: Home DNA Direct, www.homednadirect.ca (accessed June 17, 2015), which offers a ‘Weight and Wellness’ DNA test which provides information on predisposition for obesity, diet, nutrition, and eating behaviors; Genetic Center, www.genetic-center.com (accessed June 17, 2015), which offers a ‘Drug Response Gene Test’ to test an individual's response to drugs such as oral contraceptives and caffeine; See also DNA Testing Services of Canada, *Paternity Testing*, www.dnatestingcanada.com (accessed June 17, 2015), which offers a ‘Diet & Fitness Optimiziation’, which provides information on traits such as metabolism and reaction to certain foods; See also AdisonLAB Genetics, http://www.adisonlab.com/en/ (accessed June 17, 2015), which offers a ‘Food Intolerance DNA Test’.

48 See Genetic Center's ‘Child Talent Gene Test’, *supra* note 27; Map my Gene, *supra* note 27; and TheMakingsOfMe, *My Child's DNA Insights Kit*, http://www.themakingsofme.com/about-us.html (accessed June 18, 2015).

49 See, for example, White Lotus Integrative Medicine, *Weight Loss Genetic Testing | Toronto Naturopaths*, http://www.whitelotusclinic.ca/weight-loss-genetic-testing/ (accessed June 18, 2015), and Naturopathy Melbourne, *DNA Testing*, http://www.naturopathy-melbourne.com.au/dna-testing.html (accessed June 18, 2015), where they suggest that their testing service will facilitate the process of ‘[r]ealising your genetic potential through nutritional and lifestyle changes can lead to changing your genetic destiny’.

50 For a bizarre example, see Natural Health Ministries, *supra* note 4, which offers ‘homeopathic energy DNA testing’ that purports to test an individual's DNA ‘energy’ to determine how different systems in the body are functioning so that electronic homeopathy can be used to correct harmful vibrations caused by foreign substances.

51 See, for example, Town Center Wellness Chiropractic and Nutrition, http://towncenterwellness.com (accessed June 18, 2015); and McKinney Online, *Chiropractic Wellness Center Offers DNA Testing for Patient Care*, http://www.mckinneyonline.com/August-2014/Chiropractic-Wellness-Center-Offers-DNA-Testing-for-Patient-Care/ (accessed June 18, 2015). The latter clinic promises that the genetic testing will help you ‘[a]chieve your health goals with this revolutionary DNA test that compiles your DNA analysis into a report that gives you customized recommendations in: genetic health risks, fitness regimen, vitamin requirements, nutritional needs, weight management and lifestyle/behaviour’.

52 See, for example, the website of the company GeneYouIn, *How Genetic Testing and Naturopathic Medicine are Closer than You Think*, https://www.geneyouin.ca/how-genetic-testing-and-naturopathic-medicine-are-closer-than-you-think/ (accessed June 18, 2015), which explains ‘How Genetic Testing and Naturopathic Medicine are closer than you think’ and suggests that ‘Naturopathic doctors would benefit by partnering with genetic testing companies’.

53 Indeed, many alternative practices are based on scientifically questionable theories such as ‘vitalism’ (naturopaths), like cures like (homeopathy) and subluxation (chiropractors). That said, we also note there are concerns about the adequacy of medical doctors’ knowledge and understanding of genetics as well [see Sylvia Metcalfe et al., *Educating General Practitioners About Prenatal Testing Approaches and Challenges*, 25 Prenat. Diagn. 592 (2005); Shweta U. Dhar et al., *Enhancing Exposure to Genetics and Genomics Through an Innovative Medical School Curriculum*, 14 Genet. Med. 163 (2012).]

54 Brunswick Health, *DNA Testing*, www.brunswickhealth.com.au/dna-testing.html (accessed June 18, 2015). See also Nature's Intention Naturopathic Clinic, *Your Genetic Testing Center*, http://www.naturesintentionsnaturopathy.com/genetic-testing/toronto-genetic-testing-centre.htm (accessed June 18, 2015), which suggests that its genetic testing service will empower ‘[its] naturopathic doctor to modify the expression of disease years before a condition begins to develop’.

55 See, for example, Paul L. Kocken et al., *Educational Level and Attitudes Contribute to Parental Intentions About Genetic Testing for Child Obesity*, 4 J. Community Genet. 243 (2013). ‘Parents with lower levels of education and parents of non-native children were more convinced that overweight has a genetic cause and their intentions to test the genetic predisposition of their child to overweight were firmer’. See also Kristen Fischer*, New DNA Test Can Predict Childhood Obesity. Has Gene Screening Gone Too Far?*, Healthline, March 28, 2014, http://www.healthline.com/health-news/tech-genetic-test-for-obesity-newborn-gene-screenings-032814 (accessed June 18, 2015).

56 Mary E. Segal, Marcia Polansky & Pamela Sankar, *Adults’ Values and Attitudes about Genetic Testing for Obesity Risk in Children*, 2 Int. J. Pediatr. Obes. 11 (2007): ‘Even if treatment were not available, more than three-quarters believed testing should be available, more than one-third of them at birth. The youngest child age to divulge a positive result, on average, was 10 years’.

57 See Shelina Begum, *Salford Firm Launches DNA Test to Help Kids Battling Obesity*, Manchester Evening News, Jan. 8, 2014, http://www.manchestereveningnews.co.uk/news/greater-manchester-news/salford-firm-bioclinics-dna-test-6477877 (accessed June 18, 2015), where it is reported that a UK company, BioClinics, is offering obesity testing to children. ‘A DNA test could be the answer to helping to beat childhood obesity’, the article suggests, and the founder of the company, Nichola McChrystal, is quoted as saying ‘absolutely nothing is excessive when it comes to driving the [childhood] obesity trends into reverse’ and that ‘the DNA test is just one tool that can be used in the fight’. See also James Graham, *BioClinics Launches DNA Tests to Help Obese Children*, Business Desk, Jan. 8, 2014, http://www.thebusinessdesk.com/northwest/news/563051-bioclinics-launches-dna-tests-to-help-obese-children.html# (accessed June 18, 2015). It is worth noting that this is a company that also offers a special testing price for those trying to lose weight for a wedding. As suggested on the clinic website: ‘The new Weight Loss DNA Test is the perfect way to lose weight for your special day’. See BioClinics, *Need to Lose Weight for Your Wedding*, http://bioclinics.wordpress.com (accessed June 18, 2015). See also MapMyGene, *supra* note 27, which claims to be able to test 46 talents and traits in children including obesity, height, and ‘general wellness’. See also TheMakingsOfMe, *supra* note 4, which claims to be able to test a child's ‘overweight potential’ and ‘response to diet’.

58 See information pamphlet for CGC Genetics, *Genetic Testing for Obesity*, http://www.cgcgenetics.com (accessed June 18, 2015), where it is also noted, likely as a way to encourage testing, that ‘[o]besity in teenagers is becoming an important issue as it can lead to, hipercolestrolmy, hypertension and diabetes. Several studies demonstrate that infant and teenage obesity is associated with a teenager's low quality of life, especially in an emotional and scholar level’.

59 Many companies use similar rhetoric to market their testing service, suggesting that, somehow, a more tailored approach will improve outcomes. See, for example, Miller Clinic for Optimal Health, *Genetic Testing*, http://drkellymiller.com/how-we-help/genetic-testing.html (accessed June 18, 2015): ‘Take the guesswork out of finding an effective diet and exercise solution by revealing the secret steps to achieve weight goals unlocked by your specific genetics’. The White Lotus Integrative Medicine, supra note 49, suggests that ‘[w]eight loss genetic testing takes the guesswork out of weight loss!’

60 See, for example, The White House Office, *Fact Sheet: President Obama's Precision Medicine Initiative*, Jan. 30, 2015, https://www.whitehouse.gov/the-press-office/2015/01/30/fact-sheet-president-obama-s-precision-medicine-initiative (accessed June 18, 2015), and CIHR, *Harper Government Invests in Personalized Medicine*, June 2012, http://www.cihr-irsc.gc.ca/e/44825.html (accessed June 18, 2015), See also The Terry Fox Research Institute, *Federal Funding to New $15M Centre of Excellence for Personalized Medicine a Boost for TFRI's Investment in Colorectal Cancer*, Feb. 7, 2014, http://www.tfri.ca/en/NewsEvents/news/news-releases-detail/14--02--07/Federal_funding_to_new_15M_centre_of_excellence_for_personalized_medicine_a_boost_for_TFRI_s_investment_in_colorectal_cancer.aspx (accessed June 18, 2015).

61 See, for example J. Lennert Veerman, *On the Futility of Screening for Genes That Make You Fat*, 11 Plos Med., DOI:10.1371/journal.pmed.1001114 (2011), ‘testing for genetic traits that are associated with obesity makes no difference in the advice to overweight persons: increased physical activity and a healthy diet are indicated regardless of the genes’.

62 See, for example, Louise M. Aston & Mark Kroese, *Genomics of Obesity: The Application of Public Health Genomics to the Prevention and Management of Obesity in the UK*, Phg Foundation, Feb. 2013, http://www.phgfoundation.org/file/13532/ (accessed June 18, 2015); Li-Jun Tan et al., *Replication of 6 Obesity Genes in a Meta-Analysis of Genome-Wide Association Studies from Diverse Ancestries*, 9 Plos One, DOI:10.1371/journal.pone.0096149 (2014). For a review of the relevant literature, see Caulfield, *supra* note 45. See also Timothy Caulfield, *It's Not all in the Genes*, Pol'y Options, July, 2014, http://policyoptions.irpp.org/issues/old-politics-new-politics/caulfield/ (accessed June 18, 2015).

63 Miller Clinic for Optimal Health, *supra* note 59.

64 See, for example, the claim made by Peter Kay, *supra* note 9, that there is a ‘recognition that homeopathic substances can interact with the genetic blueprint and re-arrange the gene expression profile or transcriptome in human cells’.

65 Cinnamon S. Bloss et al., *Effect of Direct-to-Consumer Genome Wide Profiling to Assess Disease Risk*, 364 New Eng. J. Med. 524 (2011). See also Roger Collier, *Predisposed to Risk but not Change*, 184 Cmaj E407 (2012).

66 Timothy Caulfield, *Direct to Consumer Genetic Testing in Canada: Should We be Concerned?*, Healthy Debate, Oct 1, 2014, http://healthydebate.ca/opinions/direct-consumer-genetic-testing (accessed June 18, 2015); Theresa M. Marteau et al., *Effects of Communicating DNA-Based Disease Risk Estimates on Risk-Reducing Behaviours*, 10 Cochrane Database Syst. Rev., DOI:10.1002/14651858.CD007275.pub2 (2012). See also Christopher A. Harle et al., *Effectiveness of Personalized and Interactive Health Risk Calculators: A Randomized Trial*, 32 Med. Dec. Making 594, 603 (2012), where it was found that personalized risk estimates did not improve risk perceptions or motivate healthy behaviors.

67 Alternatively, it is possible that parents may pay less attention to fostering healthy eating and exercise habits in their children if test results indicate a low probability of becoming obese. Existing evidence on this type of behavioral response is mixed. See *id.*

68 Wagner, *supra* note 33.

69 This concern has been noted in the popular press. Emily Piccinin, *New Blood Test Predicts your Child's Athletic Future?*, Today's Parent, Feb. 13, 2014, http://www.todaysparent.com/blogs/on-our-minds/sports-blood-test/ (accessed June 18, 2015). ‘If a 10-year-old is found to be the possessor of soccer-associated gene, s/he will most likely endure a life of training specifically in that sport. What if the kid has the ability to excel at soccer but does not have the desire to play the sport?’ In addition, if a genetic test indicates a talent for a particular sport, parents may choose to restrict their children's activities to those related to that sport. Children's ability to pursue their own passions could therefore be compromised and there could be harm to the parent/child relationship by forcing children to participate in activities they do not enjoy. However, even without access to genetic information, parents may encourage or discourage their children from participating in particular activities based on a child's demonstrated abilities or body type, which may or may not align with the child's own interests and passions, giving rise to the same concern. Once again, more research will be required to determine the impact—and possible harm—of genetic information in this context.

70 Jodi T. Heshka et al., *A Systematic Review of Perceived Risks, Psychological and Behavioural Impacts of Genetic Testing*, 10 Genet. Med. 19 (2008).

71 Andrea. F. Patenaude, *Commentary: Save the Children: Direct-to-Consumer Testing of Children is Premature, Even for Research*, 36J. Pediatr. Psychol. 1122 (2011). See also Robert Klitzman, ‘*Am I my genes?’ Questions of Identify Among Individuals Confronting Genetic Disease*, 11 Genet. Med. 880 (2009) for a discussion of how genetic information in the context of disease risk may influence people's personal identities.

72 To be fair, in the context of pornography, the harms are more amorphous and contested, but, at least, their existence is supported by some empirical evidence. See, for example, Michael Flood, *The Harms of Pornography Exposure among Children and Young People*, 18 Child Abuse Rev. 383 (2009); and Paul J. Wright, *Pornography and the Sexual Socialization of Children: Current Knowledge and a Theoretical Future*, 8 J. Child. Media 305 (2014).

73 For example, the website for The Wellness Gene, http://www.wellnessgene.com/DNA-Test.html (accessed June 18, 2015), a DTC genetic testing company, suggests that ‘[k]nowing your genetic risk for these diseases gives you the opportunity to make effective and targeted lifestyle changes, whether that means increasing the frequency of certain health screenings or altering your vitamin and mineral intake’. Not surprisingly, the company also notes that they ‘offer a variety of high-quality supplements that directly support an individual's DNA weight loss and management results, and supplements that support overall health’.

74 See, for example, the allergy test provided by Accu-Metrics, *Accu-Allergy10*, http://www.accu-metrics.com/accu-allergy.php (accessed June 18, 2015), ‘intolerance is linked to various genetic factors and Viaguard's Allergy Test identifies the presence of linked antigen/antibodies’. See also Natural Health Ministries, *supra* note 4, ‘Homeopathic Energy DNA Testing’ which does not involve any form of genetic testing and instead uses electronically induced vibrations to ‘correct the vibration’ of foreign substances in an individual's hair sample.

75 For example, if a child's health is at risk as a result of a parent's refusal to administer evidence-based medicine in favor of homeopathic remedies, this may violate child welfare legislation or potentially criminal law. See CBC News*, Calgary Strep Victim's Mother ‘Will be Held Accountable,’ Say Police*, Cbc News Calgary, Nov. 23, 2013, http://www.cbc.ca/news/canada/calgary/calgary-strep-victim-s-mother-will-be-held-accountable-say-police-1.2437558 (accessed June 18, 2015).

76 See, for example, the ‘Blood Sport Food Allergy Test’ by the Calgary Centre for Naturopathic Medicine, *Testing & Diagnostics Overview*, http://www.calgarynaturopathic.com/Services/TestingOffered.aspx (accessed June 18, 2015); Family Naturopathic Clinic, *Food Allergy Testing*, http://familynaturopathicclinic.com/therapies/food-allergy-testing/ (accessed June 18, 2015); and Green Mountain Health's food allergy test that is claimed to be useful in treating everything from acne, anxiety and asthma to hyperactivity, ulcerative colitis and obesity, Green Mountain Natural Health, *Get a Food Allergy Test*, http://www.greenmountainhealth.com/naturopathic-food-allergy-test (accessed June 18, 2015).

77 Pascal Borry et al., *Legislation on Direct-to-Consumer Genetic Testing in Seven European Countries*, 20 Eur. J. Hum. Genet. 715 (2012).

78 Louiza Kalokairinou et al., *Changes on the Horizon for Consumer Genomics in the EU*, 346 Science 296 (2014).

79 Tim Caulfield, *Direct-to-Consumer Testing: If Consumers Are Not Anxious, Why are Policymakers?*, 130 Hum Genet. 23 (2011). In addition, the fact that DTC genetic tests may be regulated does not mean that it is ethical for geneticists, other scientists, or health care personnel to participate in the development, marketing or administration of these tests. To the extent that these actors are involved in these activities, words and acts addressing the ethics of their involvement may be appropriate.

80 Food and Drug Administration, June 10, 2010, letter to 23andMe, Inc., http://www.fda.gov/downloads/medicaldevices/resourcesforyou/industry/ucm215240.pdf.

81 ‘Regulations Restricting the Sale and Distribution of Cigarettes and Smokeless Tobacco to Protect Children and Adolescents’ 21 CFR Part 1140, March 19, 2010, Available from: US Government Publishing Office http://www.fda.gov/TobaccoProducts/GuidanceComplianceRegulatoryInformation/ucm360573.htm (accessed September 10, 2015).

82 Earl Howe, *Medicines and Healthcare Products Regulatory Agency: Written Question HL3347*, Parliament.uk, Dec. 16, 2014, http://www.parliament.uk/business/publications/written-questions-answers-statements/written-question/Lords/2014--12--02/HL3347 (accessed June 18, 2015); Patrice Sarrazin, *Regulatory Oversight of Genetic Testing in Canada: Health Canada Perspective*, Health Canada, July 16, 2010, http://www.ontariogenomics.ca/sites/default/files/Sarrazin.pdf (accessed June 18, 2015).

83 US Federal Trade Commission, *Docket No. C-4456, In the Matter of GeneLink, Inc., Decision and Order*, May 8, 2014, https://www.ftc.gov/system/files/documents/cases/140512genelinkdo_0.pdf (accessed June 18, 2015).

84 See David J. Kaufman et al., *Risky Business: Risk Perception and the Use of Medical Services among Customers of DTC Personal Genetic Testing*, 21 J. Genet. Counsel 413 (2012), which found that 28 per cent of survey respondents who had used DTC genetic testing discussed their test results with health care professionals. In contrast, Katherine Kolor et al., *Public Awareness and Use of Direct-to-Consumer Personal Genomic Tests from Four State Population-Based Surveys, and Implications for Clinical and Public Health Practice*, 14 Genet. Med. 860 (2012), found that one-half to three-quarters of DTC genetic test consumers studied shared their test results with health care providers.

